# Comparison of Ticagrelor and Clopidogrel for Patients Undergoing Emergency Percutaneous Coronary Intervention

**Published:** 2018-07

**Authors:** Bin YANG, Chunyan ZHENG, Haichu YU, Rui ZHANG, Shan LI, Lijuan TAN, Min LENG, Shanglang CAI

**Affiliations:** 1. Dept. of Cardiology, The Affiliated Hospital of Qingdao University, Qingdao, Shandong, 266003 China; 2. Medical Consultation Center, The Affiliated Hospital of Qingdao University, Qingdao, Shandong, 266003 China

**Keywords:** Ticagrelor, Clopidogrel resistance, Acute myocardial infarction, Thrombelastogram, Major adverse cardiac events

## Abstract

**Background:**

We compared treatments with the antiplatelets ticagrelor and clopidogrel used in patients with acute myocardial infarction (AMI) during the perioperative period for emergency percutaneous coronary intervention (PCI).

**Methods:**

A total of 120 patients were selected and randomly divided into control and observation groups (60 patients in each) from 2014–2016 at The Affiliated Hospital of Qingdao University. The patients in the control group received 300 mg clopidogrel and 300 mg aspirin for oral administration, while those in the observation group were given 180 mg ticagrelor and 300 mg aspirin orally prior to the PCI. During the operation, heparinization and a tirofiban micro-pump were used continuously.

**Results:**

Coronary artery and peripheral venous blood were extracted from each patient to obtain various parameters of thrombelastogram (TEG), and the maximum platelet aggregation rates in order to compare antiplatelet effects. Major adverse cardiac events (MACE) were recorded during the following 6-month follow-up. Analysis of the data showed no differences in terms of the time span between medication intake and stent implantation, or the dosage of heparin and tirofiban used between the two groups. Before stent implantation, and 24 and 48 h after the procedure the average R and K values of TEG in coronary artery blood and peripheral venous blood samples in the observation group were longer than those in the control group, while the α angle, MA, CI, MARAA and MARADP values were lower (*P*<0.05).

**Conclusion:**

Ticagrelor can improve antiplatelet treatment for patients with AMI during the perioperative period of emergency PCI.

## Introduction

Acute myocardial infarction (AMI) is a cardiovascular event with inherent high morbidity and mortality. Emergency percutaneous coronary intervention (PCIs) are only performed in 5%–30% of AMI cases in China (1), where anti-platelet aggregation, lipid reduction and anti-ventricular remodeling therapies are the mainstay of AMI treatment. Platelet activation plays a significant role in formation and development of AMI atherosclerotic plaques as well as during PCI treatment (2). During surgical treatment of pacemaker-induced cardiomyopathy (PICM), ischemia reperfusion injury, slow blood flow and no reflow, stunned myocardium and PCI-related infarcts closely associate with the functions of platelet activation and aggregation (3).

Clopidogrel hydrogen sulphate tablets are transformed in the body to active ingredients, which can specifically and irreversibly inhibit ADP receptors on platelet surfaces. After 24 h, the platelet inhibition rate reaches 70%–80%; after 3–7 days, a stable state is reached, with an inhibition rate of 40%–60% of platelets; then 5–7 days after withdrawal, blood platelet inhibition and bleeding time return to base line levels (4).

Experience with clopidogrel for AMI and PCI, has demonstrated that a great number of patients exhibit a serious clopidogrel resistance phenomenon, leading to low antiplatelet efficacy and the occurrence of major adverse cardiac events. The antiplatelet effect of ticagrelor was superior to that of clopidogrel, and its effects were not diminished in clopidogrel resistance patients (5).

Our study included patients with AMI who received emergency PIC in our hospital, and who received either ticagrelor or clopidogrel treatment, so as to compare antiplatelet efficacy and clinical safety of the two antiplatelet drugs during the perioperative period.

## Materials and Methods

### Object Materials

Overall, 120 patients who were diagnosed with AMI for the first time in The Affiliated Hospital of Qingdao University from October of 2014 to October of 2016 were enrolled in the study successively. All the patients met diagnostic criteria for AMI of less than 24 h of evolution, had emergency PCI indications and effective treatment expectation.

Patients excluded from the study included those complicated with other cardiac diseases (such as dilated cardiomyopathy), those requiring mechanical ventilation. Furthermore, excluded were those patients with target lesions not suitable for PCI treatment (left main coronary artery, bifurcation lesion and calcification lesions), patients with high risks of bleeding and abnormal coagulation function, or those with severe hepatic renal dysfunction.

The study was approved by the Ethics Committee of The Affiliated Hospital of Qingdao University and written informed consents were signed by the patients and/or guardians.

According to the order of admission, random numbers were assigned to divide the patients into a control group or an observation group, with 60 cases in each. In the control group, there were 38 males and 22 females, with ages ranging from 42 to 76 yr (averaging 58.7 ± 13.5 yr). There were 42 cases with ST elevation myocardial infarction (STEMI), and 18 cases with NSTEMI, the disease evolution times ranged from 1 to 20 h (averaging 10.5 ± 4.6 hours); as for target lesions, 25 cases had lesions in the left anterior descending branch, 20 in the right coronary artery, and 15 in the left circumflex artery. The coronary angiogram (CAG) showed that the degree of coronary artery stenosis (by the visual diameter method) was 85 to 99%, (averaging 94.5 ± 4.6%).

In the observation group, there were 35 males and 25 females, ranging in age between 45 and 78 yr (averaging 59.6 ± 15.7 yr). There were 40 cases with STEMI, and 20 cases with NSTEMI. The evolution time of the disease ranged from 1.5 to 18 h, averaging 11.2 ± 5.5 hours. In this group, 22 cases had lesions in the left anterior descending branch, 22 in the right coronary artery, and 16 in the left circumflex artery. The degree of coronary artery stenosis was 88–100%, averaging 95.7 ± 4.4%. Comparison of the baseline data from the two groups yielded no significant differences.

### Research Methods

The same surgery and nursing teams conducted all the PCI procedures, according to standard medical procedures. The target blood vessel flows were set as TIMI III grade. Prior to the procedure, the control group patients were administered 300 mg clopidogrel and 300 mg aspirin orally, while the observation group patients were given 180 mg ticagrelor and 300 mg aspirin instead. During the operation, heparinization and a tirofiban micro-pump were used continuously (5 μg/kg/h), and a proper stent model for each patient was chosen according to the results of coronary angiogram. After the operation, the control group patients continued taking 75 mg/d clopidogrel and 100 mg/d aspirin orally, and the observation group patients continued with 180 mg/d (90 mg bid) ticagrelor and 100 mg/d aspirin orally. Other comprehensive treatments included lipid reduction, anti-ventricular remodeling, anti-myocardial ischemia, depressurization, and blood glucose reduction therapies as appropriate.

### Parameters of Observation

Before stent implantation, coronary artery blood and peripheral venous blood samples were extracted. 24 and 48 hours after stent implantation, peripheral venous blood samples were also obtained to obtain thrombelastogram (TEG) parameters (including R value, K value, α angle, MA value and CI value) and maximum platelet aggregation rates (MARAA and MARADP, induced by arachidonic acid (AA) and adenosine diphosphate (ADP), respectively), so as to determine the occurrence rate of clopidogrel resistance (5 μmol/L ADP-induced platelet aggregation >50% was indicative of clopidogrel resistance). Follow-up of the patients was continued for 6 months after the PCI, and the incidences of major adverse cardiac events (MACE) and bleeding events were recorded.

The Thrombelastograph Analyzer TEG-5000 and supplementary reagent kaolin accelerator was bought from Haemoscope (USA) and used to perform TEGs. Among TEG parameters, the reaction time (R) points to the time required for the first fibrin clot formation (with a TEG amplitude of 2 mm) once each blood sample was placed on the analyzer; the kinetics of clot development (K) reflect the time required time for blood to clot to a certain degree (MA value 20 mm); the speed of hemagglutination formation (α angle) represents the intersection angle between the tangent line of the largest curve radian and horizontal lines; MA shows the maximum strength and hardness of the blood clot, as well as its stability; and the coagulation index (CI) <−3 signals hypocoagulability, −3 to 3 means normal coagulability and >3 means hypercoagulability.

Anticoagulant blood samples were centrifuged at 200 × g for 10 min to obtain platelet rich plasma, incubated for 10 min under 37 °C. Then either 0.5 mg/L AA or 10 μmol /L ADP were added as inducers. Light turbidimetry was applied, using Chronolog instruments.

The definition of MACE used during the follow-ups includes target vascular remodeling, recurrence of myocardial infarction, heart failure, re-hospitalization and sudden cardiac death.

### Statistical Methods

The Software SPSS20.0 (Chicago, IL, USA) was used for statistical analysis. Measurement data were presented by Mean ± Standard Deviation. For comparison between the two groups independent-sample *t* tests were performed. The comparison of data from different time points was done conducting variance analyses of repeated measurements. Enumeration data were presented by number of cases or percentages, and χ^2^ was used for comparison among groups. A *P*<0.05 was taken to indicate a statistically significant difference.

## Results

### Comparison of Intraoperative Treatment and Parameters of Implanted Stents

The times from taking the antiplatelet medication to stent implantation, the dosages of heparin and tirofiban used, the parameters of implanted stents (number of stents, lengths and diameters) were compared among the two study groups the differences found had no statistical significance (Table 1).

**Table 1:** Comparison of average intraoperative medications and parameters of implanted stents

***Groups***	***Time from Medicine Intake to Stent Implantation (min)***	***Dosage of Heparin (104 U)***	***Dosage of Tirofiban (mg)***	***Number of Stents***	***Length (mm)***	***Diameter (mm)***
Control Group	35.6 ± 6.4	2.1 ± 0.6	10.5 ± 2.4	1.2 ± 0.4	24.2 ± 4.6	18.7 ± 3.4
Observation Group	33.8 ± 6.9	2.2 ± 0.8	10.3 ± 2.1	1.3 ± 0.5	25.3 ± 4.8	17.6 ± 3.6
*t*	0.212	0.096	0.102	0.131	0.296	0.217
*P*	0.869	0.925	0.933	0.875	0.832	0.856

### Comparison of TEG Parameters and MAR Values in Coronary Artery Blood

Before stent implantation, TEG parameters like R and K values of coronary artery blood samples in the observation group were clearly prolonged, while the α angle, MA, CI, MARAA and MARADP values were dramatically decreased (*P*<0.001) the differences found between the observation and control groups had statistical significance (*P*<0.05) (Table 2).

**Table 2:** Comparison of average teg parameters and mar values in coronary artery blood samples

***Groups***	***R Value (min)***	***K Value (min)***	***A Angle (°)***	***MA Value (mm)***	***CI Value***	***MARAA (%)***	***MARADP (%)***
Control Group	6.3 ± 1.6	2.9 ± 1.1	53.2 ± 3.6	50.6 ± 4.7	−2.6 ± 1.1	59.6 ± 12.3	61.2 ± 15.2
Observation Group	7.2 ± 2.1	3.2 ± 1.3	50.8 ± 3.4	48.3 ± 5.1	−2.3 ± 1.2	55.7 ± 11.5	56.8 ± 12.4
*t*	3.659	3.524	4.122	4.065	3.632	5.623	5.527
*P*	0.035	0.038	0.024	0.026	0.033	0.008	0.011

### Comparison of TEG Parameters and MAR Values in Peripheral Venous Blood

Before stent implantation, R and K values of TEG in peripheral venous blood samples were longer in the observation group, while α angle, MA value, CI value, MARAA and MARADP values much were lower than in the control group (*P*<0.001). 24 and 48 h after stent implantation, the differences in the above parameters for the control and observation groups were maintained and the differences showed statistical significance (*P*<0.01) (Table 3).

**Table 3:** comparison of average TEG parameters and mar values in peripheral venous blood samples

***Parameters***		***Control Group***	***Observation Group***
R Value (min)	Before Stent	5.8 ± 1.5	6.2 ± 1.8
	24 h later	5.6 ± 1.6	6.0 ± 1.7
	48 h later	5.7 ± 1.7	6.2 ± 1.9
K Value (min)	Before Stent	2.6 ± 1.2	2.9 ± 1.6
	24 h later	2.5 ± 1.3	2.8 ± 1.9
	48 h later	2.5 ± 1.4	3.0 ± 1.7
α Angle (°)	Before Stent	55.5 ± 4.5	53.2 ± 4.2
	24 h later	56.7 ± 4.3	53.6 ± 4.1
	48 h later	56.2 ± 4.4	53.4 ± 4.5
MA Value (mm)	Before Stent	54.5 ± 5.2	51.2 ± 4.7
	24 h later	55.7 ± 5.3	52.3 ± 4.5
	48 h later	55.2 ± 5.6	52.1 ± 4.6
CI Value	Before Stent	2.1 ± 0.9	0.8 ± 0.5
	24 h later	2.5 ± 1.1	1.2 ± 0.5
	48 h later	2.1 ± 0.8	0.3 ± 0.4
MARAA (%)	Before Stent	85.6 ± 12.5	71.5 ± 12.4
	24 h later	91.2 ± 14.6	73.4 ± 16.3
	48 h later	87.8 ± 15.3	72.3 ± 15.5
MARADP (%)	Before Stent	82.5 ± 15.9	72.4 ± 15.7
	24 h later	84.6 ± 16.3	74.5 ± 14.3
	48 h later	83.3 ± 13.5	73.6 ± 16.2

### Comparison of Clopidogrel Resistance Incidence between the groups

In the control group, 7 patients (13.3%) were detected as being clopidogrel resistant, while in the observation group 8 cases were so (13.3%); the comparison yielded no statistically significant difference (χ^2^=0.076, *P*=0.783).

### Comparison of Safety

In the observation group, there were five patients (8.3%) with MACE; among whom one had target vascular remodeling, one experienced recurrent myocardial infarction, one developed congestive heart failure and two needed re-hospitalization. In the control group, there were thirteen cases (21.7%) with MACE; among whom two had target vascular remodeling, two had recurrent myocardial infarctions, three developed congestive heart failure, five needed re-hospitalization and one suffered sudden cardiac death. The total incidence rate of MACE in observation group was lower than that of the control group (χ^2^=4.183, *P*=0.041). None of the patients in either group experienced serious bleeding events.

## Discussion

In this study, TEG parameters like R and K values in coronary artery blood and peripheral venous blood samples from patients in the observation group were longer than those values from patients in the control group, meanwhile α angle, MA, CI, MARAA and MARADP values were lower; and all the differences were statistically significant. Comparing TEG parameters and MAR values in coronary artery blood from two groups before stent implantation was an innovative approach, which directly reflects platelet activation and local aggregation in the coronary artery, evaluating the microenvironment for PCI operation. The results of this research suggest that the platelet activation degree in the coronary artery blood was different from that in the peripheral venous blood. This could be explained by the necessity of clopidogrel to be metabolized into an active molecule by the liver cytochrome P450 (isoenzyme 2B6 and 3A4), a fact that reduces the overall bioavailability of the drug. In addition, about 40% to 50% of patients experienced serious clopidogrel resistance (6); ticagrelor, however, works directly without the need for liver metabolism and activation, and combines with the P2Y12 ADP receptor reversibly.

TEG can reflect the processes of platelet aggregation, coagulation and fibrinolysis dynamically, presented in the form of quantifiable graphics (7). The R value stands for the blood coagulation response time, the K value for the dynamic state of coagulation, the α angle for the speed of thrombosis, and the MA value is influenced by the numbers of FIB and platelets, while the CI value evaluates the whole process of coagulation. Platelet activation is closely related to thrombosis (8). The ONSET/OFFSET study showed that after 0.5 h the average inhibition rate for platelet aggregation reached 40% for a 180 mg loading dose of ticagrelor, and the same dose reached 90% inhibition (the peak value) after 2 to 4 h, while maintaining the inhibition for additional 2 to 8 h (9). Therefore, ticagrelor achieves fast and efficient platelet inhibition and should meet operation requirements for emergency PCI.

The evidence from our findings pointed to a total incidence of MACE in the observation group lower than that in the control group, while the comparison of the incidence of hemorrhaging events yielded no difference. In agreement with our findings, the PLATO study showed that compared with clopidogrel, a 12-month ticagrelor treatment further decreased the risks of cardiovascular death, myocardial infarction and apoplexy composite outcome events to 16% for patients with acute coronary syndromes (ACS), without increasing major bleeding, and reduced the cardiovascular death to 21%. Therefore, both the ESC NSTE-ACS (10) and STEMI guidelines (10, 11) recommend clopidogrel only for patients who cannot accept ticagrelor.

## Conclusion

Ticagrelor can improve antiplatelet treatment for patients with AMI during the perioperative period of emergency PCI, given its effectiveness and safety profile in comparison to clopidogrel.

## Ethical considerations

Ethical issues (Including plagiarism, Informed Consent, misconduct, data fabrication and/or falsification, double publication and/or submission, redundancy, etc.) have been completely observed by the authors.

## References

[1] Peiyuan H Jingang Y Haiyan X (2016). The Comparison of the Outcomes between Primary PCI, Fibrinolysis, and No Reperfusion in Patients ≥ 75 Years Old with ST-Segment Elevation Myocardial Infarction: Results from the Chinese Acute Myocardial Infarction (CAMI) Registry. PLoS One, 11(11): e0165672. 27812152 10.1371/journal.pone.0165672 PMC5094717

[2] Liu Y Yin H Jiang Y Xue M Guo C Shi D Chen K (2013). Correlation between Platelet Gelsolin and Platelet Activation Level in Acute Myocardial Infarction Rats and Intervention Effect of Effective Components of Chuanxiong Rhizome and Red Peony Root. Evid Based Complement Alternat Med, 2013: 985746. 23533533 10.1155/2013/985746 PMC3606717

[3] Kimmelstiel C Zhang P Kapur NK (2011). Bivalirudin is a Dual Inhibitor of Thrombin and Collagen-Dependent Platelet Activation in Patients Undergoing Percutaneous Coronary Intervention. Circ Cardiovasc Interv, 4(2): 171–179. 21364148 10.1161/CIRCINTERVENTIONS.110.959098 PMC3086141

[4] Jia LX Qi GM Liu O Li TT Yang M Cui W Zhang WM Qi YF Du J (2013). Inhibition of Platelet Activation by Clopidogrel Prevents Hypertension-Induced Cardiac Inflammation and Fibrosis. Cardiovasc Drugs Ther, 27(6): 521–530. 23887740 10.1007/s10557-013-6471-z PMC3830206

[5] Husted S Boersma E (2016). Case Study: Ticagrelor in PLATO and Prasugrel in TRITON-TIMI 38 and TRILOGY-ACS Trials in Patients With Acute Coronary Syndromes. Am J Ther, 23(6): e1876–e1889. 25830867 10.1097/MJT.0000000000000237 PMC5102280

[6] Ray S (2014). Clopidogrel resistance: The way forward. Indian Heart J, 66(5): 530–534. 25443607 10.1016/j.ihj.2014.08.012 PMC4223168

[7] Lau YC Xiong Q Ranjit P Lip GYH Blann AD (2016). Laboratory assessment of anti-thrombotic therapy in heart failure, atrial fibrillation and coronary artery disease: insights using thrombelastography and a micro-titre plate assay of thrombogenesis and fibrinolysis. J Thromb Thrombolysis, 42(2): 233–244. 26942726 10.1007/s11239-016-1344-5 PMC4912975

[8] Dobesh PP Oestreich JH (2014). Ticagrelor: Pharmacokinetics, Pharmacodynamics, Clinical Efficacy, and Safety. Pharmacotherapy, 34(10): 1077–1090. 25164528 10.1002/phar.1477 PMC4282310

[9] Toma N (2010). Randomized double-blind assessment of the ONSET and OFFSET of the antiplatelet effects of Ticagrelor versus Clopidogrel in patients with stable coronary artery disease. TheONSET/OFFSET study. Maedica (Buchar), 5(1): 75–76. 21977127 PMC3150076

[10] Silber S (2011). Immediate and Long-Term Therapy of Patients with Acute Coronary Syndromes with Thienopyridines. Current Status According to the Latest European Society of Cardiology(ESC) Guidelines. Rambam Maimonides Med J, 2(3): e0056. 23908814 10.5041/RMMJ.10056 PMC3678795

[11] Koul S Andell P Martinsson A (2014). A pharmacodynamic comparison of 5 anti-platelet protocols in patients with ST-elevation myocardial infarction undergoing primary PCI. BMC Cardiovasc Disord, 14: 189. 25516485 10.1186/1471-2261-14-189 PMC4274705

